# Extensive Alternative Splicing of KIR Transcripts

**DOI:** 10.3389/fimmu.2018.02846

**Published:** 2018-12-04

**Authors:** Jesse Bruijnesteijn, Marit K. H. van der Wiel, Nanine de Groot, Nel Otting, Annemiek J. M. de Vos-Rouweler, Neubury M. Lardy, Natasja G. de Groot, Ronald E. Bontrop

**Affiliations:** ^1^Comparative Genetics and Refinement, Biomedical Primate Research Centre, Rijswijk, Netherlands; ^2^Department of Immunogenetics, Sanquin, Amsterdam, Netherlands; ^3^Theoretical Biology and Bioinformatics, Utrecht University, Utrecht, Netherlands

**Keywords:** NK cell, killer cell immunoglobin-like receptor, KIR, human, rhesus macaque (*Macaca mulatta*), alternative splicing

## Abstract

The killer-cell Ig-like receptors (KIR) form a multigene entity involved in modulating immune responses through interactions with MHC class I molecules. The complexity of the *KIR* cluster is reflected by, for instance, abundant levels of allelic polymorphism, gene copy number variation, and stochastic expression profiles. The current transcriptome study involving human and macaque families demonstrates that *KIR* family members are also subjected to differential levels of alternative splicing, and this seems to be gene dependent. Alternative splicing may result in the partial or complete skipping of exons, or the partial inclusion of introns, as documented at the transcription level. This post-transcriptional process can generate multiple isoforms from a single *KIR* gene, which diversifies the characteristics of the encoded proteins. For example, alternative splicing could modify ligand interactions, cellular localization, signaling properties, and the number of extracellular domains of the receptor. In humans, we observed abundant splicing for *KIR2DL4*, and to a lesser extent in the lineage III *KIR* genes. All experimentally documented splice events are substantiated by *in silico* splicing strength predictions. To a similar extent, alternative splicing is observed in rhesus macaques, a species that shares a close evolutionary relationship with humans. Splicing profiles of *Mamu-KIR1D* and *Mamu-KIR2DL04* displayed a great diversity, whereas *Mamu-KIR3DL20* (lineage V) is consistently spliced to generate a homolog of human *KIR2DL5* (lineage I). The latter case represents an example of convergent evolution. Although just a single KIR splice event is shared between humans and macaques, the splicing mechanisms are similar, and the predicted consequences are comparable. In conclusion, alternative splicing adds an additional layer of complexity to the *KIR* gene system in primates, and results in a wide structural and functional variety of KIR receptors and its isoforms, which may play a role in health and disease.

## Introduction

Natural killer (NK) cells express killer-cell immunoglobulin-like receptors (KIR) that interact with major histocompatibility complex (MHC) class I molecules expressed on the cell surface of nucleated cells. Through these interactions, KIR may modulate the NK-cell activity, thereby providing regulation of the immune system in infectious diseases, pregnancy, and transplantation (1–4). KIR belong to a multigene family, which in humans comprises 17 members that are categorized into four lineages based on structure and ligand interactions; lineage I includes *KIR2DL4/5*, lineage II includes *KIR3DL1/L2/S1*, lineage III includes *KIR2DL1-3*/*2DS1-5* and the pseudogenes, and lineage V includes *KIR3DL3*. The *KIR* gene cluster is a complex entity, as is reflected by allelic polymorphism (5), gene copy number variation resulting in different haplotypic configurations (6), variegated expression (7, 8), and complex chromosomal recombination events (9–11).

The *KIR* genes are tandemly arranged on chromosome 19q13.4, each spanning 10,000–15,000 base pairs (bp), and are separated by ~1,000 bp (12). The receptors are encoded by up to nine exons, of which the first two exons encode the leader peptide, followed by exons encoding two or three extracellular Ig-like domains (2D or 3D; exons 3–5), a stem structure (exon 6), a transmembrane region (exon 7), and a cytoplasmic tail (exons 8–9) (13, 14). A long cytoplasmic tail (L) contains two immunoreceptor tyrosine-based inhibitory motifs (ITIM) and characterizes inhibitory KIR. Activating KIR feature a short cytoplasmic tail (S) and a positively charged residue in the transmembrane region, which interacts with molecules that contain the immunoreceptor tyrosine-based activation motif (ITAM).

In the past, numerous *KIR* characterization studies were mainly performed at the genomic DNA (gDNA) level, thereby lacking information about transcription and post-transcriptional modifications (PTM) of the transcripts. Recently, next-generation sequencing (NGS) has improved and speeded up characterization of the *KIR* gene cluster, resulting in the identification of novel alleles, recombinant genes, and haplotypes (9–11, 15–17). In addition, NGS also enables the characterization of transcripts that are subjected to alternative splicing. The alternative splicing of transcripts is a prevalent form of PTM, which is observed for ~95% of the human multi-exon genes, and it plays a crucial role in the regulation of protein diversity and tissue-specific gene expression (18, 19). Normally, precursor messenger RNA (pre-mRNA) is converted to mRNA by constitutive splicing, which involves the removal of introns and the ligation of exons by the spliceosome, which is a complex of five small nuclear RNAs (U1, U2, U4, U5, and U6), and multiple associated core proteins (20–22). To correctly identify the splice sites, a precise interplay of conserved sequence elements present on pre-mRNA (*cis*-acting), along with spliceosome factors (*trans*-acting) is required. Deviation from constitutive splicing, caused by variation and mutations in the pre-mRNA sequence and/or an imbalance in the *trans*-acting splice factors, can result in alternative splicing. This process can be either beneficial, as a wide variety of isoforms can originate from a single gene, or detrimental, as different isoforms can be involved in the development of various diseases, such as spinal muscular atrophy and different forms of cancer (23–25). Despite its clinical relevance, at present the splicing pattern of only a few multi-exon genes has been described thoroughly (26, 27).

Three groups reported alternatively spliced KIR transcripts that lacked complete exons (28–32). For example, *KIR2DL4* transcripts may lack exon 3 (D0 domain), suggesting the existence of a protein structure with only the D2 extracellular domain (30). Likewise, *KIR2DL5* splice variants with deletions of exon 5, exons 3 and 5, and exon 7 have been reported; these might encode protein structures lacking the extracellular D2 domain, both the D0 and D2 domains, and the transmembrane region, respectively (31). The latter suggests the existence of a soluble KIR2DL5 isoform. In addition, deletions of only fragments of the Ig-like domains have been described.

Over the past few years, the *KIR* gene family has also been characterized in non-human primate species, which provide insights into the evolution of this section of the immune system, and eventually may help to optimize and refine animal models (9, 33–37). Rhesus macaques (*Macaca mulatta, Mamu*), for example, share a close evolutionary relationship with humans, as is reflected by similar immune responses and pathologies in many models for infectious and autoimmune diseases (38–40). Although there are certain subtle differences such as different receptor lineage expansion (lineage II and III in macaques and humans, respectively), and the absence of a haplotype A and B organization in macaques, the *KIR* cluster in macaques is highly similar to that observed in humans (9, 41). Within the rhesus macaque *KIR* family, 22 genes are identified, including receptors with a single extracellular domain (*Mamu-KIR1D*), a homolog of human *KIR2DL4*, and multiple *KIR3D* gene structures (33, 42). Rhesus macaque *KIR* haplotypes can contain 4–14 genes, illustrating the extensive copy number variation (9, 33). As observed in humans, the macaque *KIR* cluster is also characterized by allelic variation, variegated expression, and chromosomal recombination.

Alternatively spliced KIR transcripts with deletions of complete or partial domain-encoding exons are described for a few *Mamu-KIR3D* genes, identified by Sanger sequencing (36, 42, 43). For example, the *Mamu-KIR3DL20* gene, which shows sequence similarity with human lineages I and V *KIR*, is hypothesized to consistently generate *Mamu-KIR2DL05* transcripts by the skipping of exon 4 (33, 42, 44, 45).

Although a few KIR splice variants were already reported in different primate species, the current literature lacks a comprehensive overview of the modifications of *KIR* genes generated by alternative splicing, as well as an indication of its possible functional consequences. By using a Single-Molecule, Real-Time (SMRT) sequencing approach on the Pacific Bioscience's (PacBio) Sequel platform, we were able to thoroughly characterize the alternative splicing of *KIR* gene transcripts in both human and rhesus macaque families. The chosen high-resolution method provides insights into the segregation of alternatively spliced KIR transcripts and the potential splicing mechanisms. The data illustrated that alternative splicing adds another layer of complexity to the KIR family in both humans and rhesus macaques. Moreover, the alternatively spliced *KIR* gene isoforms might encode receptors having a modified structure, function, and/or expression profile, which consequently might play a custom role in health and disease.

## Materials and Methods

### Transcriptome Datasets

The KIR transcriptomes of 15 related humans and 30 related rhesus macaques were reported previously (9). In addition, during the course of this study the KIR transcriptomes of another three rhesus macaque families, which in total comprised 25 macaques, and one human family, comprising six individuals, samples of whom were provided by Sanquin (Amsterdam, The Netherlands), were analyzed as previously described (9). In short, total RNA was isolated from human and rhesus macaque PBMCs, and cDNA was synthesized. Primer sets were designed for human *KIR2DL4* and *KIR2D/3D*, which amplified all human *KIR* genes except for *KIR3DL3, KIR2DL5*, and the pseudogenes. A *KIR2DL04*-specific primer set along with two *KIR2D/3D* primer sets amplified macaque *KIR*. Tagged KIR amplicons were pooled and purified, and SMRTbell libraries were generated. Sequencing was performed on a PacBio Sequel platform using P6-C4 sequencing chemistry. Informed consent was obtained from all participants.

### Identification of Alternative Spliced KIR Transcripts and Splice Elements

Subsequent to PacBio sequencing, the circular consensus sequences were selected for high read quality, and were demultiplexed based on unique barcoding. Geneious Pro R10 software (46) was used to map the PacBio reads to reference databases that included all reported full-length and partial human and rhesus macaque *KIR* allele sequences, which were derived from the IPD-KIR database and the literature (5, 33, 43, 45, 47–51), to identify 100% matched reads (0% mismatch, maximum ambiguity = 1, minimum mapping quality = 30, 80% minimum overlap identity, minimum overlap = 400). Next, the unused reads were subjected to deletion and structural variant discovery, which can align paired and unpaired reads that include structural rearrangements, deletions, and insertions, to reference sequences from the databases (0% mismatch, maximum ambiguity = 1, minimum mapping quality = 30, 10 gaps per read allowed, minimum overlap = 100). A splice variant was confirmed when observed in two or more individuals with at least three supporting reads. For each gene for which a specific splice event was confirmed, the sequence was submitted to the ENA database and received an accession number (Supplementary Tables 1, 2). In addition, Sanger sequencing was used to confirm alternative splicing in transcripts of human *KIR2DL5* (deletion of 294 bp), human *KIR2DL4* (deletion of 104/105 bp), *Mamu-KIR3DL01* (inclusion of 170 bp), *Mamu-KIR3DL20* (deletion of 300 bp and 415 bp), and *Mamu-KIR2DL04* (inclusion of 245 bp), using primers designed in the inserted region, or at the boundary of the deleted region. The skipping of exon 4 in *Mamu-KIR3DL20* transcripts was visualized by gel electrophoresis, using gene-specific primers situated at the boundary of exons 1/2 and at the end of exon 5.

### KIR Intron Sequences

Macaque *KIR* intron sequences are almost absent from the literature, except for introns in a completely sequenced rhesus macaque KIR haplotype (45). Therefore, we extracted genomic DNA (gDNA) from EDTA whole blood samples by a standard salting-out procedure, or from ~15 × 10^6^ PBMCs with an AllPrep RNA/DNA Mini Kit (Qiagen), in accordance with the manufacturer's instructions. We designed generic primer sets, tagged with PacBio barcodes, which amplified one or multiple introns, and flanking exons, of *Mamu-KIR3DL/S, Mamu-KIR1D*, and *Mamu-KIR2DL04* (Supplementary Table 3). Thermal cycling conditions were denaturation at 98°C for 2 min, followed by “x” cycles of 98°C for 20 s, “x” °C for 30 s, and 72°C for 2 min (number of cycles and annealing temperature are indicated in Supplementary Table 3 for each primer set). Sequencing was performed on a PacBio Sequel platform. The majority of the human *KIR* intron sequences were derived from the IPD-KIR database (52). Additional sequences of human *KIR* introns 6, 7, and 8, and flanking exons, were obtained by amplification with two primer sets, using the above-mentioned thermal cycling conditions (Supplementary Table 3). The obtained intron sequences could be assigned to the corresponding *KIR* genes or alleles based on the flanking exon sequences.

### Splicing Strength Prediction

Multiple prediction tools have been developed and compared to score sequence elements that are involved in splicing, such as the 3′ splice site (ss) region, including the polypyrimidine tract (PPT), and the 5′ ss (53–57). In all studies, the Maximum Entropy Modeling Scan (MaxEntScan; MES) (58), the Position Weight Matrix (PWM) via SpliceView (59), and the Human Splice Finder (HSF) (60) outperformed the other tools, and were therefore selected to predict the splicing strength of the KIR splice elements. The different prediction tools use varying nucleotide ranges to score the splicing strength of the 3′ ss, which is likely due to the degenerate nature of this motif (Supplementary Table 4). The 5′ ss was mainly defined by three exonic (−3) and six intronic nucleotides (+6). The output value of the tools also has different ranges, but a higher score always implies a more precise prediction (Supplementary Table 4). It should be noted that the scores are not a measure of effect sizes, and there are no thresholds that can predict whether or not a splice event will occur. The scores should only be used to facilitate a comparison between related splice sites. In addition to MES, PWM, and HSF, the Weight Matrix Model (WMM) (58) and NNSplice tool (61) were evaluated, and used when other tools failed to provide a splicing strength score.

## Results

### Overview of Alternative Splicing Events in Human KIR Genes

In a preceding family study, single Molecule, Real-Time (SMRT) sequencing on a Pacific Bioscience's (PacBio) Sequel platform was used to obtain *KIR2DL4* and *KIR2D/3D* transcript profiles of 15 related individuals (9). These transcript profiles partly consisted of reads that matched 100% to known *KIR* alleles. The dataset, however, also comprised a considerable number of partial sequences, and sequences that contained single nucleotide gaps. In this communication, we performed an in-depth analysis of these latter datasets, and determined that ~53 and 4% of the 100%-matched reads (error-free reads) accounted for alternatively spliced *KIR2DL4* and *KIR2D/3D* transcripts, respectively.

**Figure 1:**
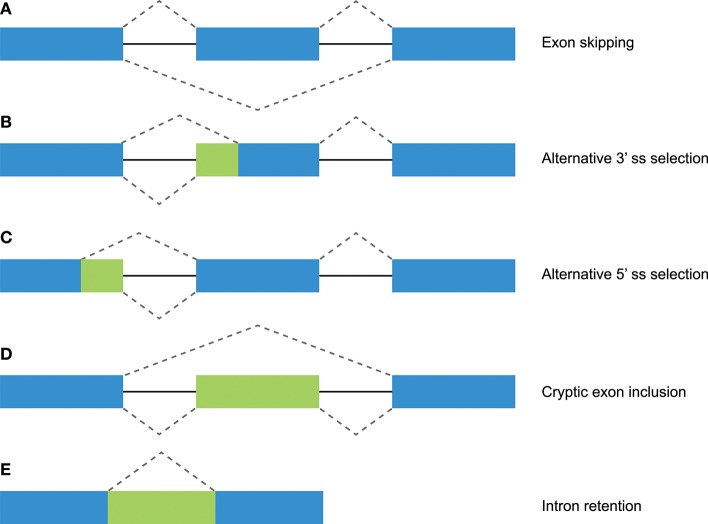
Different mechanisms of alternative splicing. Blue boxes indicate exonic regions. Green boxes indicate intronic sequences that are included in mature mRNA by alternative splicing. The upper dashed lines indicate constitutive splicing, whereas the lower dashed lines indicate alternative splicing. **(A)** Exon skipping (or “cassette exon”) is the most prevalent form of alternative splicing, which involves the complete deletion of one or multiple exons. **(B,C)** An alternative 3′ or 5′ splice site (ss) can result in a partial intron retention (observed as insertion in the mRNA) or deletion. **(D)** Intron retention is a less common form of alternative splicing, and involves the inclusion of a complete intron. **(E)** Introns can include exonic sequences that can contain intact 3′ and 5′ splice sites. The incorporation of these cryptic exons is prohibited during constitutive splicing, but they can be included into the mature mRNA by alternative splicing.

**Figure 2:**
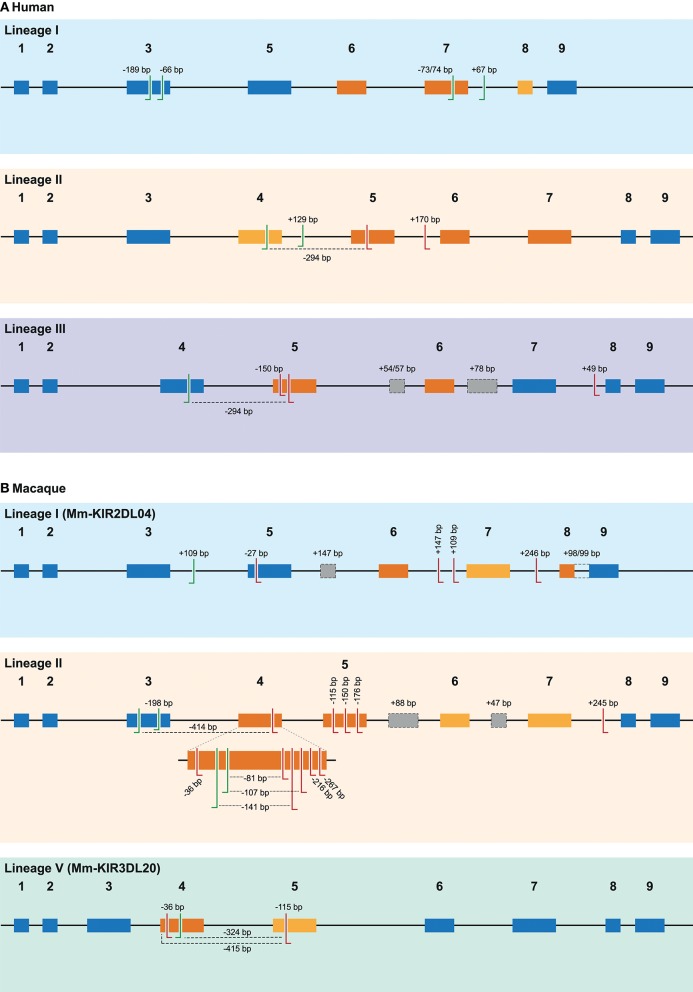
Overview of alternative splice sites in human and rhesus macaque KIR. A schematic representation of the different splice events observed in human **(A)** and rhesus macaque **(B)** KIR transcripts categorized by gene lineage. The splice events illustrated correspond to the splice events summarized in Tables 1, 2, and are indicated with the size of the inclusion (+) or deletion (–) in base pairs (bp), or by color-coding. The black line indicates the introns, whereas colored boxes represent the exons. Exons that are subjected to exon skipping are illustrated with dark orange boxes, and the exons that are only skipped in combination with one or more exons are indicated in light orange boxes. Exons that are not subjected to exon skipping are colored blue. The actual splice sites, which map to the exon/intron boundaries, have not been indicated. Alternative 3′ splice sites (ss) are indicated with green left-directed hooks, whereas alternative 5′ ss are indicated with red right-directed hooks. An alternative splice site always pair with the adjunct complement actual 3′ or 5′ splice site, except splice events that are mediated by a set of 3′ and 5′ alternative ss, which are marked with a dashed line. Cryptic exons are illustrated as gray boxes with a dashed line, and the (alternative) splice sites of these cryptic exons are not explicitly indicated. The intron retention event observed in rhesus macaque KIR2DL04 (lineage I) is indicated with a dashed line between exons 8 and 9. Exon 4 in rhesus macaque lineage II KIR genes is enlarged to more precisely illustrate the high number of alternative splice events observed.

In total, 29 distinct KIR splice events were identified (≥3 PacBio reads), of which 18 were observed in two or more related individuals (Table 1). These independently confirmed splice events involved both insertions (6 events) and deletions (12 events), and can be categorized into common types of splicing mechanisms, such as exon skipping, alternative 3′- and 5′-splice sites (ss), and cryptic exon inclusion (Figure 1 and Table 1). In Figure 2A, a schematic overview is provided of the confirmed splice events summarized in Table 1. The excision of exon 6, which encodes the stem region, represented the most frequently observed splice event, and was identified in alleles of six different *KIR* genes (Table 1). Other commonly observed splice events were the deletion of exon 5 (D2 domain), a deletion of 294 bp mediated by an alternative 5′ ss at the end of exon 4 and an alternative 3′ ss at the beginning of exon 5, an insertion of 54/57 bp following exon 5, and an insertion of 78 bp subsequent to exon 6. The remaining splice events were specific for one or two *KIR* genes. In transcripts of *KIR2DL1, KIR2DL3, KIR2DL4, KIR3DL1*, and *KIR3DL2*, at least four different splice events were observed, resulting in a diverse range of isoforms for these genes. The most diverse alternative splicing profile was observed for *KIR2DL4*, for which eight different splice events were identified. Most of these events were *KIR2DL4-*specific, including a frequently observed insertion of 67 bp subsequent to exon 7, and a deletion of 66 bp in exon 3. For transcripts encoded by activating *KIR* genes less alternative splicing events were observed, which might be explained by the lower frequency of these genes in the individuals studied. Similar splice events were observed in an additional human family comprising six individuals, confirming the obtained splice profiles, and suggesting that the data provides a comprehensive overview of alternative splicing in human *KIR*.

**Table 1:** Eighteen splice events identified in 15 human individuals by PacBio sequencing of KIR transcripts.

**Splice event**	**Deletion/inclusion**	**Size (bp)**	**Position**	**Observed in KIR genes**	**Result**
Exon skipping (“cassette” exon)	Deletion	294	Exon 5	2DL2/3/5, 3DL1	Missing D2 domain
	Deletion	51	Exon 6	2DL1/3/4, 2DS2/4, 3DL2	Missing stem region
	Deletion	104/105	Exon 7	2DL4, 3DL2	Missing transmembrane region; possible soluble receptor
	Deletion	594	Exons 4 and 5	3DL1, 3DL3	Missing D1 and D2 domains
	Deletion	155/156	Exons 6 and 7	2DL4, 3DL2	Missing stem and TM region; possible soluble receptor
	Deletion	158	Exons 7 and 8	2DL4	Missing TM region, and part of cytoplasmic tail; possible soluble receptor
	Deletion	209	Exons 6, 7, and 8	2DL4	Missing stem, TM region, and part cytoplasmic tail; possible soluble receptor
Alternative 3′ ss	Deletion	150	Start exon 5	2DL3	In-frame deletion of the first 150 bp in the D2 domain
	Inclusion	170	Following exon 5	3DL1	Stopcodon introduced
	Inclusion	49	Following exon 7	2DL1	Out-frame; stopcodon introduced
Alternative 5′ ss	Deletion	66	End exon 3	2DL4	In-frame deletion of the first 66 bp in the D0 domain
	Deletion	198	End exon 3	2DL4	Missing 66 AA in end of D0 domain. Only in combination with a deletion in the TM region.
	Inclusion	129	Following exon 4	3DL2	Stopcodon introduced
	Deletion	73/74	End exon 7	2DL4	Out-frame; stopcodon introduced
	Inclusion	67	Following exon 7	2DL4	“9A” KIR2DL4 alleles: frameshift restored ORF “10A” KIR2DL4 alleles: out-frame; stopcodon introduced
Alternative 3′ and 5′ ss	Deletion	294	Parts exons 4 and 5	2DL3, 2DS4, 3DL1/2	In-frame deletion of end D1 domain and begin D2 domain
Cryptic exon	Inclusion	54/57	Following exon 5	2DL1, 2DS1/4/5	Stopcodon introduced
	Inclusion	78	Following exon 6	2DL1/2/3, 2DS1	In-frame; positively and negatively charged residues introduced

*The events can be categorized into exon skipping (or “cassette” exons), alternative 3′ and/or 5′ splice sites (ss), and cryptic exon inclusion. The size of deletions or inclusions are indicated in base pairs (bp). The result of the alternative splice event is predicted. The background color of each splice event corresponds to the background color of the different lineages illustrated in Figure 2A*.

### *In silico* Prediction of Cis-Acting Splicing Elements

**Figure 3:**
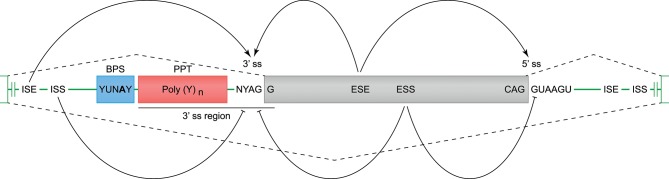
*Cis*-acting motifs that mediate constitutive and alternative splicing. The boundaries of exons (gray box) and introns (green line) are marked by splice sites (ss). At the 3′ ss, the end of the intron is characterized by an adenine and guanine (AG), and forms the basis of the 3′ ss motif. At the 5′ ss, the start of the intron is marked by a guanine and thymine (GT), and forms the basis of the 5′ ss motif (−3 bp in exon, and +6 bp in intron). Prior to the 3′ ss, a branch point sequence (BPS) and polypyrimidine tract (PPT) can be identified, and these elements are involved in spliceosome binding and intron exclusion. The 3′ splice site and PPT together are referred to as the 3′ region (−20 bp in intron, and +3 bp in exon), and can be used to predict the splicing strength. In addition to the splice site motifs, enhancer and silencer motifs can be identified in the exons (ESE, Exon Splicing Enhancer; ESS, Exon Splicing Silencer) and introns (ISE, Intron Splicing Enhancer; ISS, Intron Splicing Silencer), and can stimulate or inhibit splicing of an exon.

Constitutive and alternative splicing of pre-mRNA is regulated by *trans*-acting factors (small nuclear RNAs, spliceosome core proteins), and their cognate nucleotide sequence *cis*-elements near the intron-exon boundaries (54, 62). Essential splicing *cis*-elements are the 3′ splice site (ss), the 5′ ss, the branch point sequence (BPS), and the polypyrimidine tract (PPT) (Figure 3). Additional enhancer and silencer elements can be identified in the exons (Exon Splicing Enhancer, ESE; Exon Splicing Silencer, ESS) and introns (Intron Splicing Enhancer, ISE; Intron Splicing Silencer, ISS). These regulatory splicing sequences are degenerate, and the consensus sequences can only be loosely followed (63). Although software tools are available to predict and score the splicing strength of all different *cis*-elements (54), we mainly focused on the better modeled prediction of the splice site elements (3′- and 5′ ss, BPS, and PPT). In the following sections, different observed events (Table 1 and Figure 2A) are substantiated by the identified splice sites, and by their corresponding *in silico*-predicted splicing strength scores, per alternative splicing mechanism (Figure 1).

### Exon Skipping in Human KIR Transcripts

**Figure 4:**
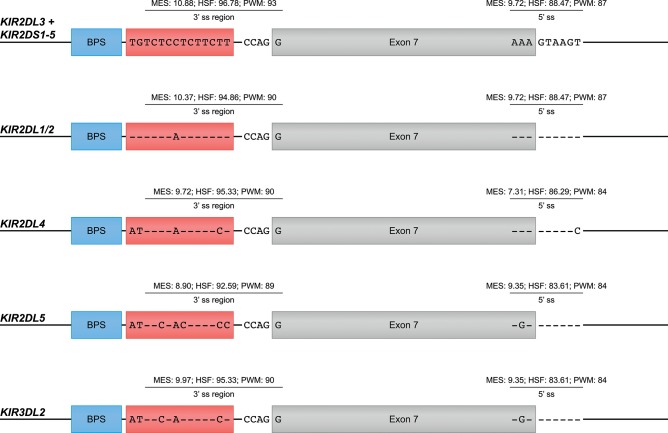
Suboptimal splice sites might mediate the skipping of exon 7 in KIR2DL4, KIR2DL5, and KIR3DL2 transcripts. Exon 7 is indicated in gray boxes, whereas the BPS and PPT elements are indicated in blue and red boxes, respectively. The 3′ ss region (3′ ss and PPT) and the 5′ ss sequences of lineage III *KIR* genes, except for *KIR2DL1/2*, are used as consensus sequences. Dashes (-) indicate sequence identity with the consensus sequence. The splicing strength scores of the 3′ and 5′ splice sites are provided. The BPS and 3′ splice sites of exon 7 were identical, but variation was observed in the PPT and 5′ splice site.

The skipping of one or multiple exons was the most frequently observed alternative splicing mechanism in the KIR transcriptomes of the human family studied (Figure 1A, Table 1, and Supplementary Table 1). The skipping of exon 7, which encodes the transmembrane region, was observed in *KIR2DL4* and *KIR3DL2* transcripts, and might be explained by variation in the splicing *cis*-elements (Figure 4). In all *KIR* genes, identical BPS and 3′ ss sequences were identified in intron 6 preceding exon 7, and were in agreement with the consensus sequences YUNAY and NYAG/G (Figures 3, 4; /marks actual splice site), respectively (64). Compared to the 5′ ss sequence of lineage III *KIR* genes (MES: 9.72; HSF: 88.47; PWM: 87), a single nucleotide variation (T/C) was observed in the *KIR2DL4* 5′ ss sequence of exon 7, and this resulted in a lower *in silico*-predicted splicing strength score (MES: 7.31; HSF: 86.29; PWM: 84) (Figure 4). Also for *KIR2DL5* and *KIR3DL2*, a decreased splicing strength score was predicted for the 5′ ss sequence of exon 7 (MES: 9.35; HSF: 83.61; PWM: 84), and these genes are discriminated from other *KIR* genes by a single nucleotide as well (A/G). Furthermore, the PPT of exon 7 in *KIR2DL1* and *KIR2DL2* contained a single adenine substitution as compared to the remaining lineage III *KIR* genes, but despite the predicted lowered 3′ ss splicing strength score, the skipping of exon 7 was not observed for the corresponding transcripts. The PPT of exon 7 of *KIR2DL4, KIR2DL5*, and *KIR3DL2*, however, varied from the lineage III *KIR* genes at four to seven nucleotide positions. This variation included the presence of two adenines that interrupted the guanine- and thymine-rich tract, and although a long continuous PPT is not required for splicing, it does appear to increase the splicing efficiency (65, 66). Indeed, a decreased splicing strength score of the 3′ ss region (3′ ss + PPT) of exon 7 in *KIR2DL4, KIR2DL5*, and *KIR3DL2* was predicted (Figure 4). Thus, the observed skipping of exon 7 in *KIR2DL4* and *KIR3DL2* transcripts might be explained by deviations in the 5′ ss and the PPT together, and suggest the existence of soluble isoforms of these receptors. In addition, the absence of exon 7 in KIR2DL4 molecules results in the loss of their activating signaling potential, which is facilitated by a positive residue in the transmembrane region. Based on the data derived from the prediction tools, the skipping of exon 7 could be expected in *KIR2DL5* transcripts as well, and was indeed reported previously (31). However, despite the presence of the *KIR2DL5* gene in some individuals, we did not identify the event in the human family studied.

Other exon skipping events involved the complex of exons 4 and 5, exon 5 only, and exon 6, respectively, encoding the extracellular domains and the stem region (Figure 2A). In particular, the skipping of exon 6 was frequently identified, and observed in *KIR* genes of lineages I, II, and III, suggesting the presence of conserved suboptimal *cis*-elements. However, between the different *KIR* genes, extensive nucleotide variation was observed in the BPS, PPT, and 5′ ss of exon 6, which resulted in a variety of predicted splicing strength scores, implying that conserved suboptimal splice sites did not mediate the splice event. In addition to less efficient splice sites, skipped exons are often characterized by longer flanking introns that can obstruct exon recognition, or that contain splice enhancer and silencer motifs (62). The two largest introns of the *KIR* gene are those flanking exon 6, and might mediate exon skipping. Phylogenetic analysis of introns 5 and 6 illustrated, however, that the introns do not have a close evolutionary relationship across the different *KIR* lineages. Despite lineage variation in the introns, ISE and ISS motifs, or elements that induce secondary intron structures, might be conserved between these introns, but these elements are hard to predict using the available *in silico* models.

### Alternative Splice Sites in Human KIR

**Figure 5:**
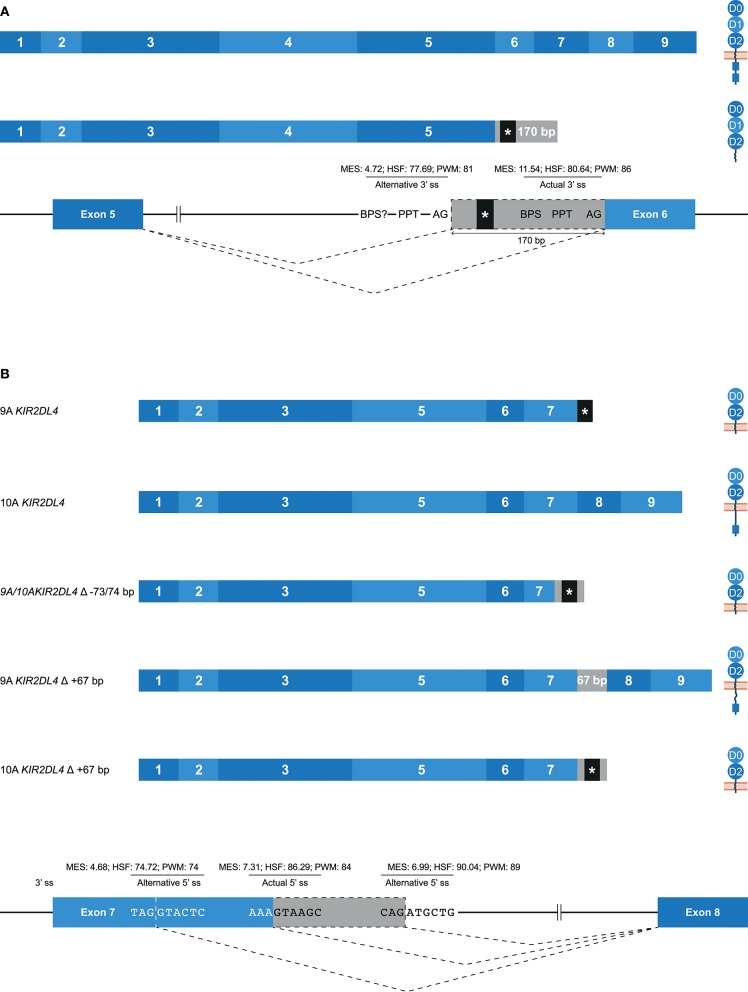
Alternative splice sites mediate deletions and insertions at the transcription level. The observed transcripts are illustrated, in which exons are indicated by blue boxes, and corresponding protein structures are schematically depicted adjacent to the transcript. Intron inclusions are indicated in gray boxes. Dashed lines indicate the potential splice events, and predicted splicing strength scores are provided for actual and alternative splice sites. Stopcodons are indicated by black boxes with an asterisk (*). **(A)** The constitutive splicing of human *KIR3DL1* results in a transcript including nine exons, and encodes a KIR3DL molecule. Alternative splicing, mediated by an alternative 3′ splice site located 170 bp downstream of the actual splice site, results in a transcript encoding only three extracellular domains. “BPS?” refers to the potential lacking of a BPS for the alternative 3′ ss. **(B)** The constitutive splicing of human “9A” and “10A” KIR2DL4 alleles results in membrane-bound molecules containing two extracellular domains, or molecules that contain two extracellular domains and a cytoplasmic tail including a single ITIM, respectively. An alternative 5′ ss located in exon 7 results in a partial deletion of the transmembrane region in both “9A” and “10A” *KIR2DL4* alleles, and the introduction of a stopcodon. A second alternative 5′ ss located in intron 7 results in a partial intron inclusion of 67 bp. In “9A” *KIR2DL4* alleles, this inclusion restores the open reading frame, and these isoforms probably express an inhibitory cytoplasmic tail. In contrast, the same splice event in “10A” *KIR2DL4* alleles results in a frame-shift that introduces a stopcodon subsequent to exon 7.

Alternative 3′ or 5′ splice sites are thought to be an intermediate between constitutively spliced and skipped exons, and can introduce in- and out-frame deletions and insertions in transcripts (Figures 1B,C, 2A) (62). An example of an alternative splice event, caused by an alternative 3′ ss, is the retention of 170 bp of intron 5, which was observed in *KIR3DL1* transcripts (Table 1). This partial intron retention introduced a premature stop codon subsequent to exon 5, resulting in a transcript that encodes only the extracellular domains, and could be explained by the presence of an additional 3′ splice site upstream of the actual splice site (Figure 5A). However, according to the *in silico* models, the splicing strength of the alternative 3′ ss region (MES: 4.72; HSF: 77.69; PWM: 81) is remarkably lower compared to the actual splice site (MES: 11.54; HSF: 80.64; PWM: 86), which might indicate that this splice event is not common. Additionally, a BPS prior to the alternative 3′ ss that matches the consensus sequence was not observed. The low number of PacBio reads (≤6 reads) for this KIR3DL1 splice variant might already be indicative that this splice event, although observed in three individuals, is not favorable over constitutive splicing. Even more, the introduction of a premature stopcodon might be indicative for the degradation of the alternatively spliced transcript by the nonsense-mediated decay. Nonetheless, the presence of this splice variant was confirmed by Sanger sequencing, and might still have functional relevance in certain NK cell subsets that are resident in specific tissues.

A partial deletion at the end of exon 7 was observed in *KIR2DL4* transcripts, and was mediated by an alternative 5′ splice site (Figure 5B). The end of exon 7 in *KIR2DL4* is marked by a poly-adenine sequence that can be nine (9A) or ten (10A) nucleotides long (30). The “9A” *KIR2DL4* alleles have a premature stopcodon subsequent to exon 7, suggesting the absence of a cytoplasmic tail, and thereby the loss of their inhibitory potential (Figure 5B). The “10A” *KIR2DL4* transcripts encode a complete receptor, including a cytoplasmic tail with a single ITIM. Deletions of 73 and 74 bp at the end of exon 7 were observed in transcripts of “9A” and “10A” *KIR2DL4* alleles, respectively (Figure 5B and Table 1). These deletions were mediated by an alternative 5′ ss that is located within exon 7, and caused a frameshift that introduced a premature stop codon, suggesting a soluble KIR2D molecule. The *in silico* models predicted that the splicing strength score of the actual 5′ ss is higher (MES: 7.31; HSF: 86.29; PWM: 84) than the splicing strength score of the alternative 5′ ss located in exon 7 (MES: 4.68; HSF: 74.72; PWM: 74), suggesting that constitutive splicing would be more prevalent. In addition, another alternative 5′ ss was observed in intron 7 of *KIR2DL4*, which resulted in a partial intron inclusion of 67 bp subsequent to exon 7. This alternative 5′ ss scored a higher predicted splicing strength (MES: 6.99; HSF: 90.04; PWM: 89) then the alternative 5′ ss located in exon 7, and even scored higher compared to the actual 5′ ss according to the HSF and PWM models. This might indicate that the inclusion of 67 bp subsequent to exon 7 in *KIR2DL4* transcripts is a prevalent splicing event, which is also supported by high PacBio read counts observed for this splice variant (an average of 115 PacBio reads per individual). In the “10A” *KIR2DL4* transcripts, the partial intron inclusion mediated by the alternative 5′ ss in intron 7 caused a frameshift that introduced a stopcodon subsequent to exon 7, and they thereby lack the cytoplasmic tail that includes an ITIM. In contrast, in the “9A” *KIR2DL4* alleles, which normally encode a truncated receptor, the open reading frame (ORF) was restored by the partial intron inclusion, resulting in transcripts that encode a KIR protein including a cytoplasmic tail. These examples suggest that alternative splicing might regulate whether the KIR2DL4 receptors contain a cytoplasmic tail, and thereby maintain their inhibitory function, or not.

### “Cryptic” Exons in Human KIR

**Figure 6:**
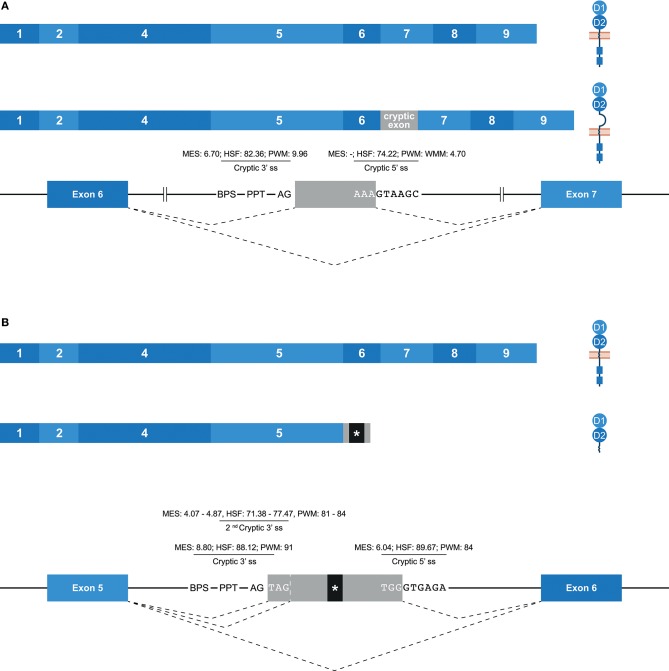
Inclusion of cryptic exons by alternative splicing. The observed transcripts are illustrated, in which exons are indicated by blue boxes, and corresponding protein structures are schematically depicted adjacent to the transcript. Cryptic exons are indicated in gray boxes. Dashed lines indicate the potential splice events, and predicted splicing strength scores are provided for cryptic splice sites. Stopcodons are indicated by black boxes with an asterisk (*). **(A)** In transcripts of KIR2DL1-3/2DS1, a cryptic exon of 78 bp was observed that originated from intron 6. This inclusion extends the region between the stem and transmembrane region by 26 amino acids, including positively and negatively charged residues. **(B)** In transcripts of *KIR2DL1* and *KIR2DS5*, a cryptic exon of 57 bp was observed, which originated from intron 5. Three bp upstream, a second cryptic 3′ ss was identified, which explained the cryptic exon inclusion of 54 bp in transcripts of *KIR2DS1* and *KIR2DS4*. At the gDNA level, the presence of one or both of these cryptic exons was also identified in other lineage III *KIR* genes and *KIR2DL5*.

Some potential exons—referred to as cryptic exons—are located within intronic regions, and are normally not spliced into mature mRNA by constitutive splicing (Figure 1E); this could be due to intrinsic defects, the presence of splice silencer elements, or the formation of inhibiting RNA secondary structures (67). Nonetheless, alternative splicing can mediate the inclusion of cryptic exons in the transcript, as is previously described for *KIR2DL1*, and this might play a role in health and disease (67–70). In the family studied, multiple alternative splice events that introduced a cryptic exon were identified. For example, an inclusion of 78 bp that originated from intron 6 was observed in transcripts of *KIR2DL1, KIR2DL2, KIR2DL3*, and *KIR2DS1* (Figure 6A). The extended transcripts remained in-frame, and the 26 introduced amino acids (cryptic exon) between the stem and transmembrane region included positively and negatively charged residues. The stretch of amino acids was found to be highly conserved in the four *KIR* gene products mentioned above, with only one single nucleotide variation present in the alleles studied. In all other *KIR* genes, except for *KIR2DL5* and *KIR3DL3*, the cryptic exon could be identified at the same position in intron 6 (~1,426 bp 3′ of exon 6, ~2,755 bp 5′ of exon 7), but sequence variability was observed, varying from 5 to 15 nucleotides, as compared to the above-mentioned conserved sequence. This variation might involve ESE and ESS motifs, which could inhibit cryptic exon inclusion. In *KIR* genes that contained the cryptic exon or the variant cryptic exon, a BPS, PPT, and 3′ ss were identified, and similar 3′ ss splicing strength scores were predicted (data not shown). The 5′ ss of *KIR2DL1-3* and *KIR2DS1* could be distinguished from the 5′ ss of other *KIR* genes by a substitution of a cytosine with an adenine, which resulted in a higher predicted 5′ ss splicing strength score in *KIR2DL1-3* and *KIR2DS1* (MES: -; HSF: 74.22; PWM: -; WMM: 4.70) compared to the *KIR* genes that had a cytosine in the 5′ ss (MES: -; HSF: 65.41; PWM: -; WMM: 1.71). Thus, this mutation might contribute to the inclusion of the cryptic exon at the transcription level. Furthermore, phylogenetic analysis illustrated that intron 6 of each *KIR* gene clustered separately, but that the evolutionary distance of *KIR2DL1, KIR2DL2, KIR2DL3*, and *KIR2DS1* was small compared to the other *KIR* genes. Although hard to predict, the variation in intron 6 sequences might involve ISE and ISS motifs that, in combination with the cryptic exon variation and 5′ ss mutation, contribute to the inclusion of the cryptic exon.

Another example of a cryptic exon inclusion is the insertion of 57 bp that was observed in transcripts of *KIR2DL1* and *KIR2DS5* (Figure 6B and Table 1). This cryptic exon originated from intron 5 (~837 bp 3′ of exon 5, ~2,259 bp 5′ of exon 6), and introduced a stopcodon subsequent to exon 5, resulting in transcripts encoding only the D1 and D2 domains. In all *KIR* genes, this cryptic exon could be identified at the same position in intron 5, with variation up to nine nucleotides. However, only in four genes (*KIR2DL1, KIR2DS1, KIR2DS3*, and *KIR2DS5*) does the cryptic exon have an intact 3′ ss region (MES: 8.80, HSF: 88.12, PWM: 91), whereas the other *KIR* genes are missing a 3′ ss at this position. Three nucleotides upstream, however, another 3′ ss could be identified in all lineage III *KIR* genes as well as in *KIR2DL5* (MES: 4.07–4.87, HSF: 71.38–77.47, PWM: 81–84), which could result in the inclusion of 54 bp subsequent to exon 5, as was observed in transcripts of *KIR2DS1* and *KIR2DS4* (Table 1). Since the predicted splicing strength score is lower in the second cryptic 3′ ss, a cryptic exon of 57 bp might be more prevalent then a 54 bp inclusion for genes that have both cryptic 3′ ss, but quantitative techniques are required to confirm this. The predicted splicing strength of the 5′ ss is similar in all genes (MES: 6.04, HSF: 89.67, PWM: 84), except for *KIR3DL2*, in which a 5′ ss was not identified. Although cryptic exon inclusion events (54 or 57 bp) were only observed in four *KIR* genes in the human family studied (Table 1), these observations suggest that the cryptic exon of 57 bp can be expected in transcripts of *KIR2DL1, KIR2DS1, KIR2DS3*, and *KIR2DS5*, whereas an identical cryptic exon of 54 bp might be observed in transcripts of all lineage III *KIR* genes and *KIR2DL5*.

### Overview of Alternative Splicing in Rhesus Macaque KIR Transcripts

In addition to the splicing profiles of human *KIR*, we also analyzed alternative splicing of *KIR* transcripts in rhesus macaques. From a preceding family-based study, macaque KIR transcriptome profiles were obtained, which consisted of 100% matched *Mamu*-*KIR* sequences (20–45%), partial sequences, and sequences that contained a single nucleotide gap (9). In-depth analysis demonstrated that ~24 and 13% of the 100%-matched *Mamu-KIR3DL/S* and *-KIR2DL04* reads (error-free reads) accounted for alternatively spliced transcripts, respectively. In total, 48 different alternative splice events were identified (≥3 PacBio reads), of which 29 were confirmed in two or more rhesus macaques (Table 2, Figure 2B, and Supplementary Table 2). To verify whether we had obtained a complete overview of the alternative KIR splicing profiles, the PacBio read coverage of some previously typed macaque transcriptomes was increased by pooling samples of three instead of 12 rhesus macaques in a single PacBio Sequel sequencing run. This resulted in an average of 40,000 PacBio reads per rhesus macaque, which is approximately four times the number of reads we obtained per macaque from the previous study. Three additional splice events were identified (≥10 PacBio reads, or confirmed in two macaques; Table 3), and although a few splice events may have been missed, this indicated that the coverage of the formerly obtained KIR transcriptomes is sufficient to provide a fairly complete overview of the alternative splicing profiles. Furthermore, three additional families, which in total comprised 25 rhesus macaques, were sequenced for their KIR transcriptome, in accordance with the previously described protocol (9). The alternative splicing profiles of the KIR transcripts in these families revealed only one novel splice event (deletion of 112 bp in exons 4 and 5), and confirmed 24 splice events that were already present in the alternative KIR splicing profiles of the formerly studied family. Moreover, three events that were previously identified in a single macaque (Table 3, events in italic) could be confirmed by analyses of the three additional families (≥3 PacBio reads, in two or more macaques). This illustrated that most, but not all, KIR splice events are shared between macaque families.

**Table 2:** Twenty-nine splice events identified in 30 related rhesus macaques by PacBio sequencing of KIR transcripts.

**Splice event**	**Deletion/Inclusion**	**Size (bp)**	**Position**	**Observed in KIR genes**	**Result**
Exon skipping (“cassette” exon)	Deletion	300p	Exon 4	3DL05, 3DL20, 3DS05	Missing D1 domain
	Deletion	294	Exon 5	3DL01/02/05/07/10, 3DL10A/3DL02	Missing D2 domain
	Deletion	51	Exon 6	2DL04	Missing stem region
	Deletion	53	Exon 8	2DL04	Missing first region cytoplasmic tail
	Deletion	594	Exons 4 + 5	3DL07, 3DL20	Missing D1 and D2 domain; possible KIR1D receptor
	Deletion	446	Exons 5, 6 and 7	3DL01/05/07/10, 3DL10A/3DL02	Missin D2 domain, stem region, and transmembrane region
	Deletion	155	Exons 6 and 7	2DL04	Missing stem and transmembrane region; possible soluble receptor
Alternative 3′ ss	Deletion	36	Start exon 4	3DL01/02/05/07/08/10, 3DL20, 3DS01/02/03/05/w08, 3DL02/3DL08A	In-frame; Missing 12 AA in the beginning of the D1 domain
	Deletion	216	Start exon 4	3DL02/08, 3DS05/w08, 3DL02/3DL08A	In-frame; Missing 72 AA in the beginning of the D1 domain
	Deletion	267	Start exon 4	3DL05, 3DS05	In-frame; Missing 89 AA in the beginning of the D1 domain
	Deletion	27	Start exon 5	2DL04	In-frame; Missing 9 AA in the beginning of the D2 domain
	Deletion	115	Start exon 5	3DL07/08/10, 3DL20, 3DS03, 3DL02/3DL08A, 3DL10A/3DL02	Out-frame; Stopcodon introduced
	Deletion	150	Start exon 5	3DL02/w03/10	In-frame; Missing 50 AA in the beginning of the D2 domain
	Deletion	176	Start exon 5	3DL07, 3DL10A/3DL02	Out-frame; Stopcodon introduced
	Inclusion	109	Following exon 7	2DL04	Out-frame; Stopcodon introduced
	Inclusion	147	Following exon 7	2DL04	Stopcodon introduced
	Inclusion	245/246	Following exon 7	2DL04, 3DL07	Stopcodon introduced
Alternative 5′ ss	Deletion	198	End exon 3	3DL02, 3DS05, 3DL02/3DL08A	In-frame; Missing 66 AA in the end of the D0 domain
	Inclusion	109	Following exon 3	2DL04	Stopcodon introduced
Alternative 3′ and 5′ ss	Deletion	81	Within exon 4	3DS02	In-frame; Missing 27 AA in center of the D1 domain
	Deletion	107	Within exon 4	3DS02	Out-frame; Stopcodon introduced
	Deletion	141	Within exon 4	3DL08, 3DSw08, 3DL02/3DL08A	In-frame; Missing 47 AA in center of the D1 domain
	Deletion	414	Parts exons 3 and 4	3DS05	In frame; Missing parts D0 and D1 domains
	Deletion	324	Parts exons 4 and 5	3DL20	In frame; Missing parts D1 and D2 domains
Exkon skipping + Alt. 3′ ss	Deletion	415	Exon 4 and start exon 5	3DL20	Out-frame; Missing the D1 domain and stopcodon introduced
Cryptic exon	Inclusion	88	Following exon 5	3DL01	Out-frame; Stopcodon introduced
	Inclusion	147	Following exon 5	2DL04	Stopcodon introduced
	Inclusion	47	Following exon 6	3DL07	Out-frame; Stopcodon introduced
Intron retention	Inclusion	98/99	Following exon 8	2DL04	“98 bp” alleles: out-frame; Stopcodon introduced “99 bp” alleles: in-frame; 33 additional AA′s subsequent to exon 8

*The events are listed per alternative splicing mechanism. The size of deletions or inclusions are indicated in base pairs (bp). The result of the alternative splice event is predicted. The background color of each splice event corresponds to the background color of the different lineages illustrated in Figure 2B*.

**Table 3:** Three rhesus macaque samples were pooled for a PacBio Sequel sequencing run, which provided a four-fold coverage compared to the obtained transcriptome profiles.

**Splice event**		**Deletion/Inclusion**	**Size (bp)**	**Position**	**Observed in KIR genes**	**Result**
High coverage	Alt. 5′ ss	Deletion	209	End exon 4	3DL20	Out-frame; Stopcodon introduced
	Alt. 3′ ss + Alt. 5′ ss	Deletion	48	In exon 5	3DL05, 3DL08, 3DS02	In-frame; Missing 16 AA in the beginning of the D2 domain
	Exon skipping	Deletion	345	Exons 5 + 6	3DL20	Missing the D2 domain and stem region
Additional families	*Alt. 3′ ss + Alt. 5′ ss*	*Deletion*	*97*	*In exon 4*	*3DL02, 3DSw08, 3DSw09*	*Out-frame; Stopcodon introduced*
	*Alt. 3′ ss*	*Deletion*	*79*	*Start exon 5*	*3DL10A/3DL08, 3DL11*	Out-frame; Stopcodon introduced. 3DL11*009: ORF restored in cytoplasmic tail
	Alt. 3′ ss + Alt. 5′ ss	Deletion	112	Parts exons 4 + 5	3DL07, 3DL11	Out-frame; Stopcodon introduced
	*Exon skipping*	*Deletion*	*645*	*Exons 4 + 5+ 6*	*3DL05, 3DL20*	*Missing D1 and D2 domains, and stem region*

*Three additional splice events were confirmed (≥10 PacBio reads, or confirmed in two macaques). Also, the alternative splicing profiles of three additional families, consisting of 25 rhesus macaques in total, were characterized. Only a single splice event was identified that was not observed in the main dataset, whereas three previously observed, but not confirmed, splicing events (italics) were confirmed by the additional rhesus macaque families*.

### Common Alternative Splicing Events in Rhesus Macaque KIR

As in human KIR, all independently confirmed splice events observed in rhesus macaque *KIR* could be categorized into common alternative splicing mechanisms (Figure 1), which are listed in Table 2 and schematically illustrated in Figure 2B. Splice events that involved *Mamu-KIR1D* are not included in this table and schematic figure, and will be discussed separately in the following section. The deletion of the first 36 bp of exon 4 was the most frequently observed alternative splice event, and is mediated by a conserved alternative 3′ ss that is present in most *Mamu-KIR1D/3D* alleles (Table 2). The predicted splicing strength score of the alternative 3′ ss (MES: 8.24–10.27, HSF: 90.11–90.53, PWM: 88–92) was higher (HSF and PWM), or similar (MES), compared to the predicted score of the actual 3′ ss (MES: 8.25–10.20, HSF: 86.61–88.52, PWM: 84–87). Transcripts with this in-frame deletion lacked 12 amino acids at the start of the D1 domain, including three positively charged residues that might be involved in protein folding or ligand binding. In exon 4 of most *Mamu-KIR3D* genes, two other alternative 3′ ss could be identified, resulting in transcripts with in-frame deletions of 216 and 267 bp (Figure 2B and Table 2). Likewise, four alternative 3′ ss could be identified in exon 5 of most *Mamu-KIR3D* genes, of which the one that mediated an out-frame deletion of 115 bp was most frequently observed. Other common alternative splice events observed in rhesus macaque KIR involved the skipping of exon 5 and the deletion of 446 bp (exons 5, 6, and 7). *Mamu-KIR1D, -KIR3DL20*, and *-KIR2DL04* displayed a remarkable alternative splicing profile, and will be discussed in more detail in the next sections.

### Extensive Alternative Splicing in *Mamu-KIR1D*

**Figure 7:**
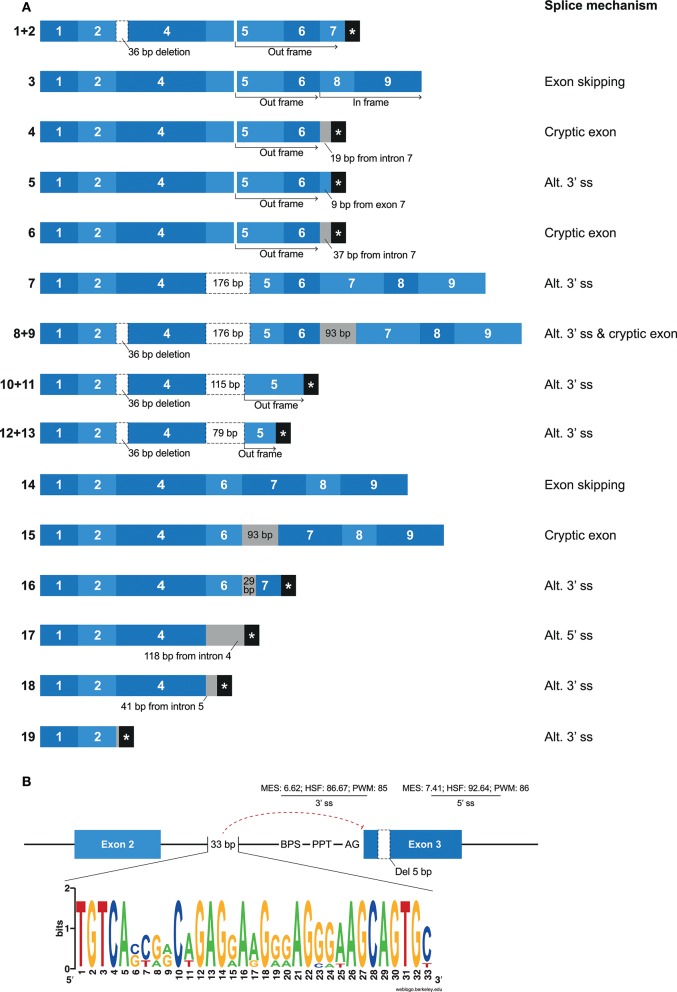
Overview of alternative splicing in *Mamu-KIR1D*. **(A)** Nineteen different *Mamu-KIR1D* transcripts were observed; each transcript is illustrated by blue boxes per exon. White boxes with a dashed outline indicate partial exon deletions, and intronic inclusions are indicated in gray boxes. Multiple transcripts have an out-frame region, due to a deletion of 7 bp in exon 5 at the gDNA level, which can introduce a stopcodon (#1, 2, 4–6). However, the complete inclusion of exon 5 combined with the skipping of exon 7 can restore the open reading frame (#3). The partial deletion of exon 5 might result in Mamu-KIR1D molecules containing an intact inhibitory cytoplasmic tail (#7–9), or in molecules that only consist of a single D1 domain (#10–14). Transcripts that completely skipped exon 5 might encode membrane-bound molecules including an inhibitory cytoplasmic tail (#14, 15), or molecules that only encode the D1 domain, with or without the stem region (#16–18). A second variant was observed for some transcripts, which involved the deletion of 36 bp at the beginning of exon 4, which was also observed in lineage II KIR genes (#1/2, 8/9, 10/11, 12/13; Table 2). **(B)** Exon 3 is present at the gDNA level in *Mamu-KIR1D*, but none of the KIR1D isoforms contain the D0 domain encoded by this exon. The BPS, PPT, and both 3′ and 5′ splice sites of exon 3 are intact, just as in human lineage III *KIR* genes, and predicted splicing strength scores are provided. Intron 2 of all lineage III *KIR* genes, including *Mamu-KIR1D*, lack 33 bp in intron 2 compared to all other *KIR* genes. The lack of this 33 bp stretch might inhibit constitutive splicing of exon 3, as indicated by the red dashed line. The weblogo plot shows the nucleotide sequence composition of this 33 bp stretch that is present in all *KIR* genes except the KIR lineage III genes, which might mediate the constitutive splicing of exon 3.

*Mamu-KIR1D*, which is the only lineage III *KIR* gene in rhesus macaques, was identified in ~25–30% of the defined *Mamu*-KIR haplotypes, and, so far, only three different alleles are reported, suggesting a high level of conservation at the exon level (9). Hershberger and colleagues described nine different splice variants using Sanger sequencing (43). In our KIR transcriptome profiles obtained by PacBio sequencing, we identified a complex array of nineteen different alternatively spliced *Mamu*-*KIR1D* transcripts that originated from a single allele (*Mamu*-KIR1D^*^002) (Figure 7A). Up to 15 different *Mamu-KIR1D* isoforms could be identified in a single individual (≥3 PacBio reads), which illustrates extensive alternative splicing. These splice variants could be explained by exon skipping, alternative 3′- and 5′ ss, and cryptic exons. At the genomic DNA (gDNA) level, three domain-encoding exons could be identified, but only exon 4 (D1 domain) was present in all transcribed *Mamu*-*KIR1D* isoforms. On the basis of gDNA analysis, it was revealed that exon 3 of *Mamu-KIR1D* contained a 5 bp deletion (71), and was constitutively skipped, similar to what is observed in human lineage III *KIR* genes. However, an intact BPS, PPT, 3′ ss (MES: 6.62, HSF: 86.67, PWM: 85), and 5′ ss (MES: 7.41, HSF: 92.64, PWM: 86) could be identified for exon 3 of *Mamu-KIR1D*, which may suggest that another mechanism plays a role in the constitutive skipping of this exon. An explanation might be the absence of 33 bp in intron 2, which characterizes all lineage III *KIR* genes in both humans and macaques (Figure 7B). This intron part is a purine-rich element that might be essential for spliceosome binding, and leads in its absence to the exclusion of exon 3 at the transcription level.

In most of the identified *Mamu-KIR1D* isoforms, exon 5 was present or partially included (Figure 7A, #1–13). Due to a 7 bp deletion in exon 5 at the gDNA level, complete inclusion of this exon at the mRNA level by constitutive splicing resulted in a frameshift that introduced a stopcodon in the beginning of exon 7 (Figure 7A; #1, 2). The remaining transcripts that included exon 5 either skipped exon 7, but had in-frame exons 8 and 9 (Figure 7A; #3), or had intronic/exonic inclusions subsequent to exon 6 that introduced a stopcodon (Figure 7A; #4–6). These transcripts probably encode soluble and truncated KIR1D receptors, respectively. In other *Mamu-KIR1D* isoforms, the first part of exon 5 was skipped, which resulted in in-frame transcripts that encoded the D1 domain, the second part of the D2 domain, and an intact cytoplasmic tail (Figure 7A; #7–9), or out-frame transcripts that had a stopcodon subsequent to exon 5 (Figure 7A; #10–13). Transcripts that completely lacked exon 5 encoded membrane-bound isoforms with the D1 domain and an ITIM-containing cytoplasmic tail (Figure 7A; #14, 15), or isoforms that encoded only the D1 domain (Figure 7A; #16–18). The deletion of 36 bp at the beginning of exon 4, as is observed in most lineage II *Mamu-KIR* genes (Table 2), was also identified in multiple Mamu-KIR1D transcripts; these isoforms could appear with and without this splice event (Figure 7A; #1/2, 8/9, 10/11, 12/13).

In other Old World monkey species such as cynomolgus macaques (*Macaca fascicularis*), olive baboons (*Papio anubis*), and vervet monkeys (*Chlorocebus aethiops*), orthologs of *Mamu-KIR1D* were observed that also displayed the 5 and 7 bp deletions in exons 3 and 5, respectively (36, 71). In addition, a comparison of intron 2 of these genes revealed that they also lack the purine-rich element of 33 bp, which might explain the constitutive skipping of exon 3. It is not known whether these orthologs are also subjected to extensive alternative splicing. In humans, no orthologs of *Mamu-KIR1D* were identified. However, multiple human *KIR2DS4* alleles that skip exon 3—and that have a 22 bp deletion at the gDNA level in exon 5, which introduces a frameshift that resulted in an early stopcodon subsequent to exon—have been described as *Mamu-KIR1D* analogs (72).

### Consistent Alternative Splicing of *Mamu-KIR3DL20*

**Figure 8:**
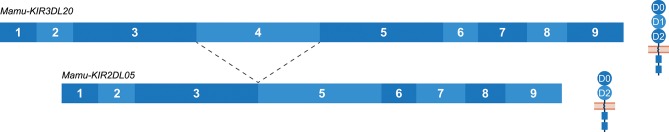
The skipping of exon 4 in *Mamu-KIR3DL20* transcripts to generate *Mamu-KIR2DL05* transcripts. Transcripts are illustrated with the exons indicated in blue boxes, and corresponding protein structures are schematically depicted adjacent to the transcript. The splice event is indicated with dashed lines. Exon 4 (D1 domain) is consistently skipped for all studied *Mamu-KIR3DL20* alleles to generate *Mamu-KIR2DL05* transcripts.

The *Mamu-KIR3DL20* gene is present on most, but not all, reported *Mamu*-KIR haplotypes, and has been considered a framework gene. Phylogenetic analysis has illustrated a relationship between *Mamu-KIR3DL20* and human linage I (*KIR2DL4, KIR2DL5*) and V (*KIR3DL3*) *KIR* genes (45, 71). Indeed, exon 3 of *Mamu-KIR3DL20* showed similarity to human *KIR2DL5*, and exons 4 and 5 displayed similarity to human *KIR3DL3*. The exons encoding the cytoplasmic tail of *Mamu-KIR3DL20* are more related to macaque *KIR* genes. Multiple studies have suggested that the frequently identified *Mamu-KIR2DL05* transcript is a splice variant of the *Mamu-KIR3DL20* gene, in which exon 4 (D1 domain) is spliced out (33, 43, 44, 71, 73). Our results substantiates that *Mamu-KIR2DL05* is indeed a splice variant of *Mamu-KIR3DL20*, and that this splice event is consistent for every identified *Mamu-KIR3DL20* allele in the rhesus macaques studied (Figure 8). In addition, gel electrophoresis indicates that the amount of exon 4 skipping in Mamu-KIR3DL20 transcripts is considerable (Supplementary Figure 1). The 3′ ss region of exon 4 in *Mamu-KIR3DL20* is intact (MES: 8.25, HSF: 86.96, PWM: 85), although the predicted splicing strength is slightly lower compared to the 3′ ss of most other macaque *KIR* genes (MES: 9.84, HSF: 88.52, PWM: 85). Due to a single substitution of a cytosine with a thymine, the 5′ ss of exon 4 in *Mamu-KIR3DL20* alleles (MES: 6.95, HSF: 94.52, PWM: 88) also scored lower compared to the remaining *KIR* genes (MES: 9.22, HSF: 96.51, PWM: 92). Therefore, these suboptimal splice sites might contribute to the skipping of exon 4 in *Mamu-KIR3DL20*, resulting in *Mamu-KIR2DL05* transcripts. Moreover, the skipping of exon 4 might be mediated by intron 3 of *Mamu-KIR3DL20*, which is 450–650 bp shorter compared to intron 3 of other *Mamu-KIR* genes. This may result in modified or missing splicing elements in intron 3, thereby influencing the spliceosome efficiency. Of note is that in human *KIR3DL3*, which has an exon 4 similar to *Mamu-KIR3DL20*, intron 3 is also shorter, but the consistent skipping of exon 4 is not reported for this human gene. In contrast to exon 4 in all reported human and macaque *KIR* genes, exon 4 of *Mamu-KIR3DL20* is completely conserved in all 22 reported rhesus macaque alleles. This observation suggests selective pressure, and indicates an important function of exon 4 (D1 domain) in the recognition of Mamu-KIR3DL20 receptors, or in the formation of Mamu-KIR2DL05 splice variants via splicing enhancer or silencer motifs.

The consensus sequence of the *Mamu-KIR2DL05* transcripts, generated by the alternative splicing of the *Mamu-KIR3DL20* gene, showed 89.5% similarity to the consensus sequence of constitutively spliced human *KIR2DL5* transcripts, and suggests a convergent evolution of this gene in humans and macaques. Although the exact mechanism and function of the consistent skipping of exon 4 in *Mamu-KIR3DL20* resulting in *Mamu-KIR2DL05* transcripts is not completely understood, it does illustrate that alternative splicing in macaques can introduce a second *KIR2DL* transcript additional to *Mamu-KIR2DL04*. As well as the skipping of exon 4, *Mamu-KIR3DL20* transcripts that lacked exons 4 and 5 (594 bp deletion; Table 2) were also frequently observed. These transcripts were not consistently observed in all macaques, however, and seem to encode inhibitory receptors with a single extracellular domain (D0).

### Alternative Splicing in *Mamu-KIR2DL04* Is Mainly Gene-Specific

**Figure 9:**
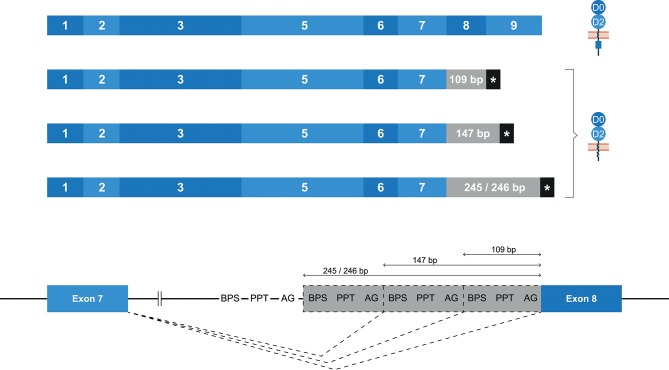
Alternative splice sites in intron 7 of *Mamu-KIR2DL04*. Transcripts are illustrated with the exons indicated in blue boxes, and corresponding protein structures are schematically depicted adjacent to the transcript. Intronic inclusions are indicated in gray boxes, and stopcodons are marked by a black box with asterisk (*). Splice events are indicated with dashed lines. Three alternative 3′ splice sites were identified in intron 7 of *Mamu-KIR2DL04*, and result in intron inclusions of 109, 147, and 245/246 bp subsequent to exon 7. All these inclusions result in a frameshift that introduces a stopcodon, indicating membrane-bound KIR2D receptors without an inhibitory cytoplasmic tail.

Whereas, human *KIR2DL4* is a framework gene, the macaque ortholog *Mamu-KIR2DL04* is identified on ~65–75% of the reported *Mamu*-KIR haplotypes (9, 33). As with *KIR2DL4* in human, the most diverse splicing profile in macaques was observed for *Mamu-KIR2DL04*, including 10 confirmed splice events, of which 9 were gene-specific (Table 2). Exon skipping events were observed in exons 6–8. In *Mamu-KIR2DL04* transcripts, the skipping of exon 7 is only observed in combination with the skipping of exon 6 (155 bp in total) (Figure 2B). The skipping of exon 8, which encodes a part of the cytoplasmic tail, was only observed in Mamu-KIR2DL04^*^015. In two macaques that expressed this allele, no complete transcripts were identified, indicating allele-specific consistent exon skipping. Other events involved alternative splice sites, of which three were located in intron 7, which resulted in partial intron retentions subsequent to exon 7 (Figure 9 and Table 2). These intronic insertions introduced a stopcodon, and the three corresponding transcripts probably encode a membrane-bound receptor that lacks a cytoplasmic tail. Similar alternatively spliced transcripts were observed for human “10A” *KIR2DL4* alleles with an insertion of 67 bp subsequent to exon 7 (Figure 5B). However, whereas in human *KIR2DL4* the splice event is mediated by an alternative 5′ ss, resulting in an inclusion of the first part of intron 7, in macaques the intron inclusions originate from the end of intron 7, and are mediated by alternative 3′ splice sites. Notably, three out of the four alternative splice sites in intron 7 could be identified in the human and macaque *KIR2DL4* orthologs, but no similar alternatively spliced transcripts were shared between the species.

## Discussion

The plasticity of the *KIR* genes is manifested by allelic polymorphism, copy number variation, the expansion and contraction of haplotypes, variegated expression, and the generation of hybrid genes by recombination. Here we demonstrate that alternative splicing adds an additional layer of complexity by the generation of isoforms originating from a single *KIR* gene. This phenomenon appears to be a structural aspect of the *KIR* gene cluster in different primate species. In total, 18 human and 55 macaque splice events were documented (Figures 2, 7A and Tables 1–3), including gene-specific events, and events that were observed across different *KIR* lineages. The potential mechanisms that mediate these splice events were categorized into common types of alternative splicing, and the responsible motifs were predicted and scored with different software tools. Overall, the current overview of the *KIR* splicing profiles in humans and rhesus macaques indicates that the generation of different KIR isoforms might be of functional relevance in health and disease.

Alternative splicing can diversify the characteristics of the encoded protein, including the domains it contains, its ligand interactions, cellular localization, and signaling properties. As a result, different isoforms encoded by the same gene can execute distinct functionalities. The receptor structure, and to a lesser extent the functional characteristics, of some KIR isoforms could be predicted based on the alternatively spliced KIR transcripts. The skipping of one or multiple exons that encode the extracellular domains (exons 3–5) likely results in the formation of KIR1D or KIR2D receptors, which can have distinct binding properties compared to the constitutively spliced isoform. A frequently observed splice event in human *KIR* is the skipping of exon 6, which encodes the stem region. The function of this region is not yet clear, but it might be involved in the flexibility and orientation of the receptor. Crystallography and *in vitro* lysis assays illustrated, for instance, that the stem region is not involved in ligand binding, but may contribute to the inhibitory signaling function (74, 75). Therefore, isoforms that lack the stem region might be less stringent in delivering inhibitory signals. Transcripts that completely or partially lack exon 7 probably encode soluble receptors, whereas the absence of exons 8 and/or 9 indicates a loss of inhibitory signaling function. The consequences of events facilitated by splice mechanisms other than exon skipping is harder to predict. For example, the in-frame deletion of 36 bp in exon 4, which is observed in 14 different *Mamu-KIR* genes and mediated by an alternative 3′ ss, might result in a different D1 domain orientation, in distinct ligand interactions, or in an aberrant folding of the complete receptor. The conservation of this alternative 3′ splice site may indicate a selective pressure on a functional characteristic of the KIR isoform generated by this splice event. *In vitro* binding and inhibition assays with KIR isoform-transfected cells could elucidate the function of these less predictable splice variants. However, it should be noted that a large proportion of the splice events may not result in functional receptors and these product may be subjected to the nonsense-mediated decay pathway, as was previously reported in human proteomic studies (76–78). This non-productive splicing is likely a redundancy relating to the rapid evolution of splice sites, from which beneficial isoforms are positively selected, although it has also been suggested that non-functional splicing is a mechanism to downregulate expression of the protein encoded by constitutive splicing (79, 80). Nonetheless, the number of splice events that we identified (Figures 2, 7A and Tables 1–3), together with the observed segregation of splice events and the sharing of splice mechanisms resulting in similar consequences on protein level in humans and rhesus macaques, suggests that at least a part of the alternative splicing profiles contributes to a structural and functional variety of KIR receptors.

All common alternative splicing mechanisms were observed in human and rhesus macaque *KIR* genes, except for intron retention, which was only observed in a single splice event in *Mamu-KIR2DL04*. In both species, similar exon skipping events were observed (Figure 2 and Tables 1–3), although in humans more events involved the skipping of multiple exons, especially exon 7 and its flanking exons. In addition to most exon skipping events, only the deletion of 198 bp in the end of exon 3, which is mediated by an alternative 5′ ss, was shared between humans and rhesus macaques. In macaques, this deletion was observed in three different genes, whereas in humans this splice event was specific for *KIR2DL4*, and was only observed in combination with a second deletion in the transmembrane region. These isoforms probably have an aberrant D0 domain, which might result in modified binding properties. All other splice events were only identified in one of the two species. In contrast to human KIR splice events, most splice events in macaque KIR involved the domain-encoding exons. This could be related to the expansion of lineage II *KIR* genes in macaques, which contain three extracellular domains, and therefore might have more flexibility to modify the domains without compromising ligand binding.

The skipping of exon 4 (D1 domain) was consistently observed in transcripts of *Mamu-KIR3DL20* (lineage V), which is considered a framework gene in rhesus macaques, and resulted in Mamu-KIR2D transcripts that only encoded the D0 and D2 domains. These alternatively spliced transcripts seem to be a functional analog of human *KIR2DL5* (lineage I) and they share a similarity of 89.5%, suggesting a convergent evolution of this structure. In macaques, the *Mamu-KIR2DL05* transcripts were identified in all individuals, whereas in humans, *KIR2DL5* is only present on specific haplotypes (group B haplotypes). This is an indication that the alternatively spliced *Mamu-KIR2DL05* transcripts, or the *Mamu-KIR3DL20* gene itself, are essential to rhesus macaques. The complete conservation of exon 4 in all *Mamu-KIR3DL20* alleles further supports this. Exon 4 in *Mamu-KIR3DL20* seems to be essential in facilitating its own consistent skipping, or in the interaction of Mamu-KIR3DL20 molecules, and might therefore be conserved by selective pressure. This conserved character is not observed for any other exon in human or macaque *KIR*. The skipping of exon 4 in *Mamu-KIR3DL20* transcripts illustrates how alternative splicing can expand the plasticity of the KIR repertoire by generating isoforms of two different *KIR* lineages from a single gene.

The skipping of exon 5, and exons 4 and 5 together, was observed in *KIR* genes of humans and macaques, and the events might have similar consequences. The skipping of exon 3 was not observed in human and macaque lineage I and II *KIR* genes, which implies essential properties for the D0 domain. It has been reported that the D0 domain is involved in the direct binding of MHC class I molecules (81), whereas others described only a modulatory role for this domain in KIR3D receptors (82, 83). Furthermore, the cell surface level of human KIR3DL1 could be modified by D0 polymorphisms, such as the substitution of a valine with a leucine at position 18 (V18L), which prevents the surface expression of KIR3DL1^*^053 (84). The characteristics of the D0 domain might be essential for KIR3D function in both species, and therefore the alternative splicing of this domain might be subjected to negative selection.

A large number of alternative splicing events were observed for the *Mamu-KIR1D* gene (Figure 7A), which is the only lineage III *KIR* gene in macaques and is highly conserved, as only three alleles have been documented in the apparent functional sections of the gene (exons 1, 2, and 4). Despite an intact BPS, PPT, and 3′ and 5′ splice sites, exon 3 is constitutively skipped in this gene. An explanation for this phenomenon can be found in the absence of a purine-rich stretch of 33 bp in intron 2 of *Mamu-KIR1D* (Figure 7B), which is also lacking in human lineage III *KIR* genes. Human and macaque *KIR* genes, which do include exon 3 in their transcripts, have an intron 2 that contains the purine-rich 33 bp. Within these genes, the 33 bp appear highly conserved, which might indicate its essential role for spliceosome recognition. Furthermore, the *Mamu-KIR1D* gene shows extensive alternative splicing subsequent to exon 4, and this might be due to the introns flanking exons 5 and 6. In rhesus macaques, introns 5 and 6 of lineage II *KIR* genes are ~2,000 and 914 bp in length, respectively. Introns 5 and 6 of *Mamu-KIR1D* (lineage III) are ~3,290 and 4,330 bp, respectively, and similar intron lengths are observed in human lineage III *KIR* genes. In humans, lineage III *KIR* genes mainly interact with HLA-C molecules. In rhesus macaques, however, no homolog of HLA-C is identified, and most *Mamu*-KIR probably interacts with members of the expanded repertoire of MHC-A and -B molecules. Therefore, selective pressure to conserve the lineage III gene might be low, which makes the large introns observed for Mamu-KIR1D prone to mutations that might induce alternative splicing. As such, an initial macaque KIR2D gene is now translated into Mamu-KIR1D. At present it is unclear whether any of the different identified Mamu-KIR1D isoforms are functional in macaques.

The extent of the impact that alternative splicing has on the KIR repertoire is dependent not only on which splice variants are formed but also on the frequency of the splice events. In this study, the PacBio Sequel platform was used to determine the alternative splicing profiles, but this method does not provide quantification, and may only be used as a quantitative indication. Approximately 4 and 53% of the 100%-matched human KIR2D/3D and KIR2DL4 reads accounted for alternatively spliced transcripts. In rhesus macaques, 24 and 13% splice variants were obtained from the 100%-matched KIR2D/3D and KIR2DL04 PacBio reads, respectively. These percentages indicate abundant alternative splicing for human *KIR2DL4* and macaque *KIR2D/3D*, but one has to be cautious with the interpretation of these numbers, as the quantification value of the PacBio platform is low. Furthermore, preferential amplification of the used primer sets cannot be ruled out, which may also have an effect on the calculated numbers. More reliable quantification methods, like droplet digital PCR (ddPCR) or RNA-seq, are hard to adapt on a multigene family such as KIR. Therefore, we are currently only able to provide quantitative indications of alternative KIR splicing.

In this study, and in other studies that reported KIR isoforms (28–30, 36, 42, 43), the splice variants were identified in whole blood samples, and might therefore give a representation of the complete splicing profile. However, tissue-specific alternative splicing has been reported, and suggests isoforms with a local specialized function (85). Especially in the brain, testis, and liver, increased alternative splicing events can be identified, which mainly involved exon skipping and alternative splice sites. NK cells that reside in tissues are reported in multiple organs, such as the intestines, lungs, liver, spleen, lymph nodes, brain, eye retina, and uterus, and can be phenotypically and functionally distinct from the NK cells in peripheral blood (86–93). The diversity of NK-cell subsets in the different tissues includes selective expression of KIR, but might also involve distinct alternative splicing profiles of the *KIR* genes. The regulation of tissue-specific alternative splicing is complex, and involves the differential expression of splicing factors and epigenetic modifications, such as methylation and histone acetylation (94–96). In tissues that should dampen the immune response to avoid inflammation, like the eye retina, or tissues that require high immune surveillance, like the intestines and liver, alternative splicing might provide the required isoforms. Also in the uterine tissue, phenotypically and functionally distinct NK cells (uNK cells) have been identified that mainly express *KIR2DL4*, and are involved in pregnancy. This NK cell subset can interact with the highly expressed HLA-G molecules in the uterus, which are subjected to alternative splicing, to maintain the fetal-maternal interface and induce cytokine production (97–99). Alternative splicing might modify the activity and interactions of KIR2DL4 expressed on the uNK cells, for example, by skipping the transmembrane region to generate soluble KIR2DL4 receptors (Figure 4), or by an insertion of 67 bp that can regulate the presence of an inhibitory cytoplasmic tail (Figure 5B).

Overall, we characterized the alternative splicing profiles of *KIR* genes in human and macaque families, which provides an illustration of the potential formation of protein isoforms. These posttranscriptional modifications might contribute to the complexity of the *KIR* gene family of both species, human and macaque, and result in a wide structural and functional variety of receptors that might be involved in health and disease.

## Author Contributions

JB, MvdW, NdG, NO, and AdV-R performed all practical work. JB wrote the manuscript. NL provided human samples. NGdG and RB supervised the project and edited the manuscript.

### Conflict of Interest Statement

The authors declare that the research was conducted in the absence of any commercial or financial relationships that could be construed as a potential conflict of interest.

## Supplementary Material

Supplementary Figure 1 Visualization of alternative splicing in Mamu-KIR3DL20 transcripts by gel electrophoresis. In the left lane marker bands are shown (top to bottom: 5,000, 2,000, 850, 400, 100 bp), and in the right lane the PCR products of Mamu-KIR3DL20 are shown, using primers that were designed at the boundary of exons 1/2 and at the end of exon 5. From top to bottom, the product bands correspond with the constitutively spliced Mamu-KIR3DL20 transcript, the Mamu-KIR2DL05 transcript (exon 4 skipped), and the transcript that was subjected to the excision of 415 bp (exon 4 and the first 115 bp of exon 5). The lowest band shows aspecific amplification. The sequences were confirmed by Sanger sequencing.

Supplementary Table 1 Eighteen different splice events were observed for human KIR. For each *KIR* gene for which a splice event was observed, an accession number is listed.

Supplementary Table 2 Twenty-nine different splice events were observed for rhesus macaque KIR. For each *KIR* gene for which a splice event was observed, an accession number is listed.

Supplementary Table 3 Primer sets to amplify human and macaque intron sequences. In human, two generic primer sets amplified most KIR2D genes. In macaques, different primer sets were used to amplify certain regions of different KIR genes.

Supplementary Table 4 The Maximum Entropy Modeling Scan (MaxEntScan; MES) (68), the Position Weight Matrix (PWM) via SpliceView (69), and the Human Splice Finder (HSF) were used to predict most splicing strenght scores. If these tools failed to provide a splicing strenght score, the Weight Matrix Model (WMM) (68) and NNSplice tool (71) were used. The definition of the 3′ and 5′ splice site could differ between the different models and are listed: + determine the number of nucleotides within the exon, and -display the number of nucleotides within the intron. Also, the models define different score ranges, in which a higher value always implies a better predicted splice site. Only the HSF model provided scores for the branch point sequence (BPS), exonic splice enhancers (ESE), and exonic splice silencers (ESS).
