# Supplementary figures and images for: Extensive Alternative Splicing of KIR Transcripts
Source: Front Immunol. 2018 Dec 4;9:2846. doi: 10.3389/fimmu.2018.02846 (PMC6288254; doi:10.3389/fimmu.2018.02846)

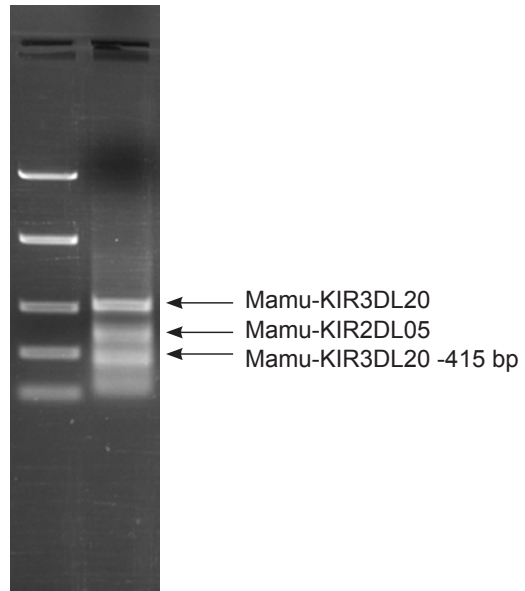

Supplement: Supplementary Figure 1 — Visualization of alternative splicing in Mamu-KIR3DL20 transcripts by gel electrophoresis. In the left lane marker bands are shown (top to bottom: 5,000, 2,000, 850, 400, 100 bp), and in the right lane the PCR products of Mamu-KIR3DL20 are shown, using primers that were designed at the boundary of exons 1/2 and at the end of exon 5. From top to bottom, the product bands correspond with the constitutively spliced Mamu-KIR3DL20 transcript, the Mamu-KIR2DL05 transcript (exon 4 skipped), and the transcript that was subjected to the excision of 415 bp (exon 4 and the first 115 bp of exon 5). The lowest band shows aspecific amplification. The sequences were confirmed by Sanger sequencing. [file Data_Sheet_1.PDF]
